# The Predictive Role of Extracellular NAPRT for the Detection of Advanced Fibrosis in Biopsy-Proven Non-Alcoholic Fatty Liver Disease

**DOI:** 10.3390/ijms24021172

**Published:** 2023-01-07

**Authors:** Angelo Armandi, Giorgia Colombo, Chiara Rosso, Gian Paolo Caviglia, Antonella Olivero, Maria Lorena Abate, Marta Guariglia, Nuria Perez Diaz del Campo, Gabriele Castelnuovo, Davide Giuseppe Ribaldone, Giorgio Maria Saracco, Armando A. Genazzani, Elisabetta Bugianesi

**Affiliations:** 1 Department of Medical Sciences, University of Turin, 10126 Turin, Italy; 2 Metabolic Liver Research Program, University Medical Center, Department of Internal Medicine I, Johannes Gutenberg University, 55131 Mainz, Germany; 3 Department of Pharmaceutical Sciences, Università del Piemonte Orientale, 28100 Novara, Italy

**Keywords:** non-alcoholic fatty liver disease, liver fibrosis, non-invasive tests, NAMPT, NAPRT, visfatin, non-alcoholic steatohepatitis, oxidative stress

## Abstract

Intrahepatic oxidative stress is a key driver of inflammation and fibrogenesis in non-alcoholic fatty liver disease (NAFLD). We aimed to investigate the role of extracellular Nicotinamide phosphoribosyltransferase (eNAMPT) and extracellular nicotinic acid phosphoribosyltransferase (eNAPRT) for the detection of advanced fibrosis. eNAMPT and eNAPRT were tested in 180 consecutive biopsy-proven NAFLD patients and compared with liver stiffness (LS) and the FIB-4 score. eNAMPT was similarly distributed across fibrosis stages, whereas eNAPRT was increased in patients with advanced fibrosis (*p* = 0.036) and was associated with advanced fibrosis (OR 1.08, *p* = 0.016). A multiple stepwise logistic regression model containing significant variables for advanced fibrosis (eNAPRT, type 2 diabetes, age, male sex, ALT) had an area under the curve (AUC) of 0.82 (Se 89.6%, Sp 67.3%, PPV 46.7%, NPV 93.8%) when compared to that of LS (0.79; Se 63.5%, Sp 86.2%, PPV 66.0%, NPV 84.8%) and to that of the FIB-4 score (0.73; Se 80.0%, Sp 56.8%, PPV 44.9%, NPV 86.6%). The use of eNAPRT in clinical practice might allow for the better characterization of NAFLD patients at higher risk of disease progression.

## 1. Introduction

Non-alcoholic fatty liver disease (NAFLD) represents the most frequent form of chronic liver disease [1] and is tightly linked to the diverse features of metabolic syndrome (mainly visceral obesity and type 2 diabetes [T2DM]). Simple fat accumulation (NAFL—non-alcoholic fatty liver) is a relatively benign condition, first-line storage of either the excessive flow of free fatty acids from peripheral adipose tissue or newly synthesized (de novo lipogenesis) in the presence of increased exogenous sugar intake. The enhanced lipid oxidation and intrahepatic deranged pathways of insulin resistance in the picture of metabolic syndrome bring NAFL to a pro-inflammatory environment with increased oxidative stress, necro-ptosis, and parallel fibrogenesis process [2]. This form of liver injury, called non-alcoholic steatohepatitis (NASH), is a chronic, progressive entity that may lead to more advanced forms of liver disease, including cirrhosis, portal hypertension, and hepatocellular carcinoma [3].

Liver fibrosis represents the most relevant prognostic factor in the NAFLD population, and in particular, advanced fibrosis has been widely linked to long-term adverse outcomes [4]. Currently, a liver biopsy is still needed for the identification and staging of liver fibrosis. Multiple non-invasive tools are being investigated to replace or reduce liver histology in clinical practice; so far, liver stiffness (LS) by transient elastography and Fibrosis-4 (FIB-4) score are the most widely used and recommended in the last European Guidelines [5].

Nicotinamide phosphoribosyltransferase (NAMPT) and nicotinic acid phosphoribosyltransferase (NAPRT) are intracellular enzymes involved in the biosynthesis of nicotinamide adenine dinucleotide (NAD) [6]. Both enzymes have been extensively studied and are now considered pharmacological targets in cancer and in immune-mediated inflammatory disorders. In fact, their inhibition interrupts the energy NAD supply to cancerous and immune cells [7,8,9]. It is now well established that both enzymes are moonlighting proteins, i.e., they possess distinct functions other than acting as mere catalysts. In detail, these proteins can be released in the extracellular milieu and act as cytokines (referred to in this context as eNAMPT and eNAPRT).

Ample evidence exists in the literature showing increased plasma levels of eNAMPT in inflammatory conditions [10,11]. Less evidence exists for eNAPRT, but a similar situation is expected since eNAPRT is strongly enhanced in acute inflammatory diseases, including sepsis, hence driving inflammatory responses related to the activation of macrophages [12]

Based on the mechanistic role of eNAMPT and eNAPRT in the inflammatory and oxidative stress environment, the aim of this study was to explore the accuracy of these enzymes for the identification of advanced fibrosis in individuals with biopsy-proven NAFLD.

## 2. Results

### 2.1. Characteristics of the Study Cohort

Baseline characteristics of the study cohort are shown in Table 1.

We retrospectively included 180 consecutive patients with biopsy-proven NAFLD. The median age was 47.0 [38.0–58.0] years, and the male sex represented 63.3% of the total. The median body mass index (BMI) was 29.2 [26.3–32.9] kg/m^2^, and T2DM was present in 27.8% of the patients. The median LS was 7.4 [5.8–10.0] kPa, while the median FIB-4 score was 0.9 [0.6–1.3]. Median eNAMPT levels were 4.1 [1.8–8.2] ng/mL, and median eNAPRT levels were 2.1 [0.9–5.1] ng/mL. At liver histology (Table 2), NASH was detected in 77.8% of cases, while advanced fibrosis was present in 30.6% of cases.

**Figure 1:**
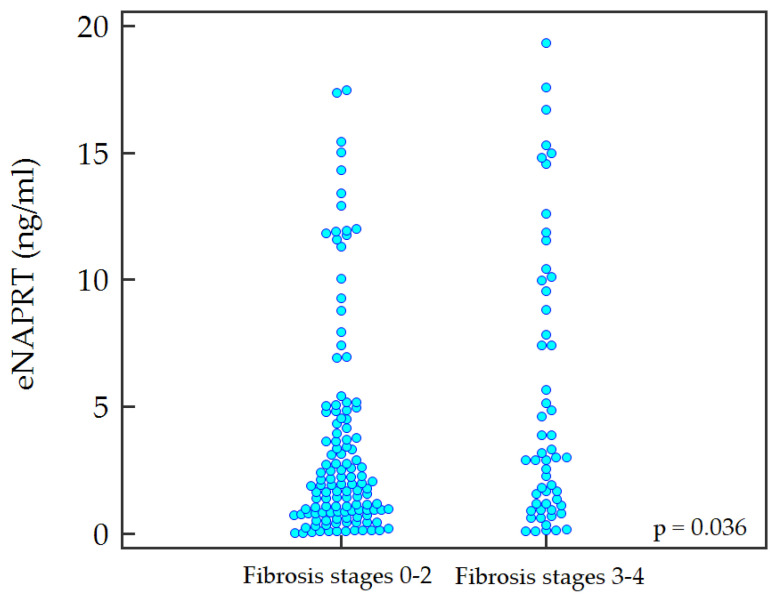
Distribution of extracellular serum nicotinate phosphoribosyltransferase (eNAPRT) according to fibrosis stages.

Serum levels of eNAPRT were significantly higher in patients with advanced fibrosis: with a median of 1.9 [0.8–4.6] ng/mL in stages 0–2 of fibrosis, with respect to 3.0 [1.1–9.4] ng/mL in advanced fibrosis (*p* = 0.036) (Figure 1).

No differences were detected with regard to eNAPRT levels and the presence of NASH. Similarly, serum eNAMPT levels were similarly distributed across all fibrosis stages and with respect to the presence of NASH (Supplementary Figure S1).

### 2.2. Predictive Ability of Non-Invasive Tests and eNAPRT for Advanced Fibrosis

In univariate logistic regression analysis (Table 3), eNAPRT was associated with advanced fibrosis (OR 1.08 [95% CI 1.01–1.15], *p* = 0.016). Similarly, both the LS and FIB-4 scores were associated with advanced fibrosis: OR 1.33 [95% CI 1.20–1.49], *p* < 0.0001, and OR 2.40 [95% CI 1.44–3.97], *p* < 0.0001.

At the receiving operator curve (ROC) analysis (Table 4), eNAPRT showed an area under the curve (AUC) of 0.60 for a Youden index of 2.76 ng/mL (Se 56.4%, Sp 64.0%, PPV 40.8%, NPV 76.9%). The AUC for LS was 0.79 for a cut-off of 9.4 kPa (Se 63.5%, Sp 86.2%, PPV 66.0%, NPV 84.8%), while AUC for the FIB-4 score was 0.73 for a cut-off of 0.86 (Se 80.0%, Sp 56.8%, PPV 44.9%, NPV 86.6%).

### 2.3. Multiple Stepwise Regression Model for the Identification of Advanced Fibrosis

To improve the accuracy of eNAPRT in the prediction of advanced fibrosis, we built a multiple stepwise regression model, including eNAPRT > 2.76 (by Youden index), together with the following variables: age, type 2 diabetes, male sex, ALT, AST, BMI, gamma-glutamyl transferase, total cholesterol, high-density lipoprotein (HDL) cholesterol, and triglycerides (Table 5).

At multivariate analysis, the following variables were significantly associated with advanced fibrosis: eNAPRT (OR 2.73 [95% CI 1.20–6.16], *p* = 0.015); age (OR 1.03 [95% CI 1.01–1.07], *p* = 0.049); T2DM (OR 3.49 [95% CI 1.63–9.05], *p* = 0.004); male sex (OR 2.84 [95% CI 1.63–9.05], *p* = 0.002); ALT (OR 1.01 [95% CI 1.00–1.02], *p* = 0.021).

The resulting model had the following formula: y = 1.004 × (eNAPRT > 2.76 y/n) + 0.037 × (age in years) + 0.011 × (T2DM y/n) + 1.346 × (male sex y/n) − 6.191.

### 2.4. Receiving Operator Curve Analysis of the Model for the Identification of Advanced Fibrosis and Comparison with Other Non-Invasive Tests

**Figure 2:**
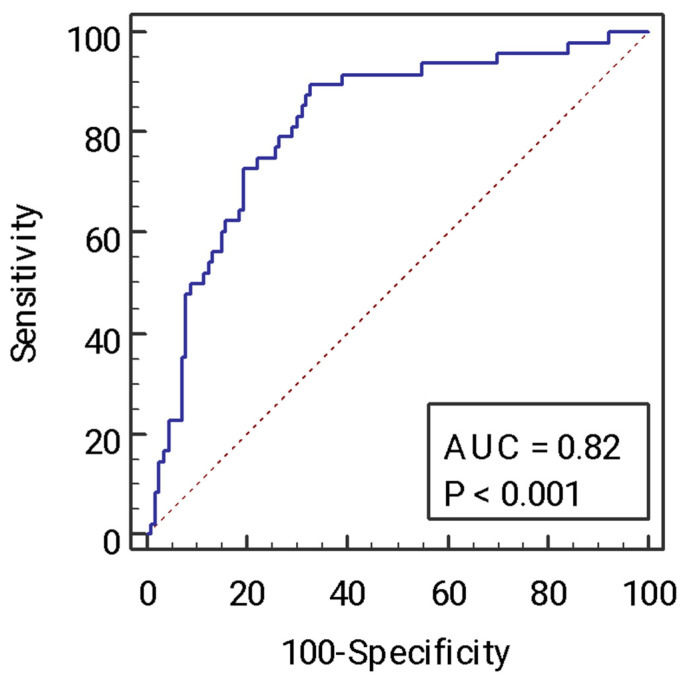
Area under the curve (AUC) of the model of advanced fibrosis.

The model had an AUC of 0.82 for the identification of advanced fibrosis (Figure 2) for a Youden index of 0.52 (Se 89.6%, Sp 67.3%, PPV 46.7%, NPV 93.8%), *p* < 0.001.

**Figure 3:**
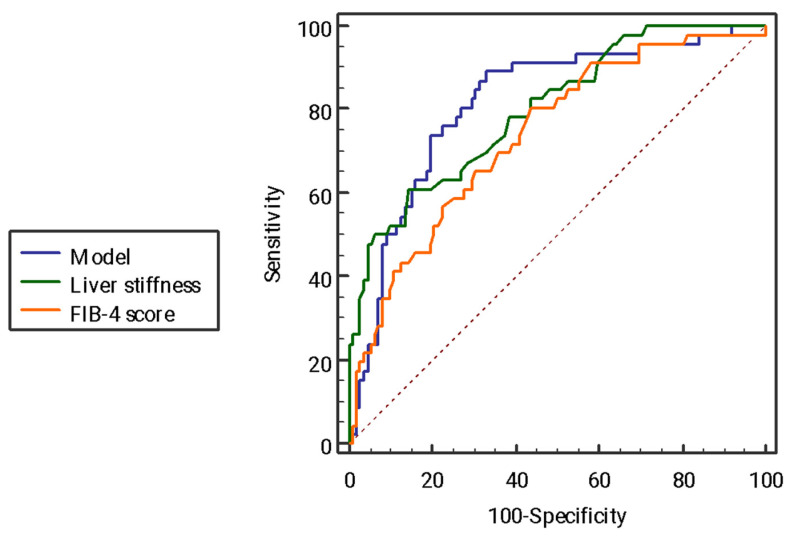
Comparison of receiving operator curves for the prediction of advanced fibrosis. Abbreviation. FIB-4: fibrosis-4.

At pairwise AUC comparisons, no difference was found between the model and LS (difference 0.01, DeLong *p* 0.734). Similarly, no difference was found between the AUC of the model with that of the FIB-4 score (0.07, DeLong *p* = 0.104) (Figure 3).

## 3. Discussion

In this cross-sectional, retrospective study of consecutive biopsy-proven NAFLD patients, we found a significant association of eNAPRT with advanced liver fibrosis. A model, including NAPRT, age, sex, and T2DM, reached a good accuracy for the identification of advanced fibrosis, similar to that of the LS and FIB-4 score, and with excellent NPV.

Intrahepatic oxidative stress is the main driver of liver injury inside the spectrum of NAFLD. The multiple sources of damage deriving from the metabolic syndrome, including insulin resistance, visceral obesity, excessive intake of saturated fats, and fructose-sweetened beverages (inside the paradigm of the “Western lifestyle”), ultimately lead to a hepatocyte proinflammatory environment [13]. Repeated damages, reactive oxygen species arising from the incomplete oxidation of excessive intrahepatic fatty acids, and the activation of Kupffer cells from pro-inflammatory signals of visceral adipose tissue insulin resistance [14] are common sources of oxidative stress. In particular, hepatic insulin resistance is able to promote liver inflammation through the activation of the innate immune system and production of harmful cytokines [2], including tumor necrosis factor-alpha, interleukin-1beta, interleukin 6, and nuclear factor kappa-light-chain-enhancer of activated B cells (NF-kB) pathways. Similarly, the proinflammatory environment promotes and worsens insulin sensitivity, favoring the progressiveness of NASH and metabolic inflammation [15]. Eventually, these stimuli activate quiescent hepatic stellate cells into metabolically active myofibroblasts, responsible for the increased scar tissue deposition and advanced stages of liver disease [16].

The identification of advanced fibrosis represents the main challenge in the NAFLD clinical setting, given its crucial role in prognostication. Risk stratification according to the progression of liver disease is warranted, and multiple strategies to overcome the limitations and invasiveness of liver biopsy are under prolific investigation [17].

In this setting, the identification of enzymes acting as biomarkers of oxidative stress might play a crucial role in NAFLD. In particular, intracellular NAMPT overexpression and increased eNAMPT levels were described in several metabolic and inflammatory disorders, including obesity, T2DM, atherogenic inflammatory diseases, and inflammatory bowel disease [10], while molecular mechanisms associated with intracellular NAPRT expression and eNAPRT secretion are less known. In addition, eNAMPT has been long recognized to be identical to the Pre-B cell enhancing factor (PBEF), first described for its ability to synergize with interleukin-7 and stem cell factors, increasing the number of pre-B cell colonies [18] and to visfatin: an adipokine, released from adipose tissue. In the extracellular space, NAMPT then exerts a pro-inflammatory cytokine activity [19,20,21]. In macrophages, eNAMPT promotes cell survival upon ER stress, up-regulates MMP-9 and MMP-2, and is able to induce the M2-polarization of macrophages in leukemia [10]. There is some data, albeit scarce, on eNAPRT that shows that this protein also induces the polarization of macrophages [12].

So far, this is the first study to address the potential role of eNAPRT in NAFLD fibrosis staging. Most studies have been conducted on eNAMPT across the spectrum of NAFLD. Several studies have shown that serum eNAMPT levels are elevated in NAFLD, correlating it with portal inflammation [22] and the stage of fibrosis [23]. However, a recent meta-analysis of 21 studies involving 1923 individuals showed that, despite the limited quality of the included studies, no association was found between eNAMPT and either histological features of NASH or the fibrosis stages [24]. This evidence is consistent with our findings, where similar levels of eNAMPT were detected with regard to both NASH and fibrosis.

Interestingly, in our cohort, increased levels of eNAPRT were significantly and independently associated with advanced fibrosis, along with T2DM, which is at the top of metabolic disease drivers in NAFLD. These results may be potentially linked to the mechanistic role of intracellular NAPRT in systemic inflammation, where its overexpression was mainly associated with a negative prognosis in cancer [25]. Furthermore, eNAPRT was shown to be present in the extracellular space and in the sera of patients with sepsis or septic shock due to bacterial infections, relating it to acute inflammatory conditions [12].

In this study, an eNAPRT-containing model for advanced fibrosis had the highest AUC when compared to other non-invasive tools, displaying the best negative predictive value (above 93%). This model includes simple and readily available variables that can be exploited for a tailored selection of candidates for liver biopsy.

This study has some limitations that need to be addressed. The retrospective nature of the study did not allow for further investigation, and all analyses could be performed only on the available data. The results are also burdened by the relatively small sample size from a single center, hence limiting their generalizability. The lack of significance in the eNAMPT levels across the different features of NAFLD did not allow for further investigation of its role in advanced fibrosis. In addition, the lack of a validation cohort limits the robustness of the model. However, the main strength of this study is the well-characterized cohort of biopsy-proven NAFLD patients with concomitant blood samples and LS that could allow for reliable comparisons.

In conclusion, in patients with biopsy-proven NAFLD, increased eNAPRT levels are significantly associated with advanced fibrosis. On the contrary, no significant differences in eNAMPT levels were observed with regard to either fibrosis stages or the presence of NASH. A predictive model, including eNAPRT, for the identification of advanced fibrosis, could be used in clinical settings for a better characterization of patients at higher risk for fibrosis progression and more advanced stages of liver disease.

## 4. Materials and Methods

### 4.1. Study Population

This retrospective, cross-sectional study involved 180 consecutive patients who underwent liver biopsy from 2011 to 2019 at the Division of Gastroenterology and hepatology of the University of Turin for the suspicion of NAFLD. Other etiologies for the liver disease were excluded, including chronic viral hepatitis (hepatitis C or hepatitis B virus infection), cholestatic, autoimmune, genetic-based, iron, and copper storage disorders. Individuals undergoing hepatotoxic or steatogenic medications were excluded. Significant alcohol intake was excluded according to the weekly thresholds (210 gr/week for males and 140 gr/week for females) through administered questionnaires. Only features of NAFLD were detected in liver histology. No clinical, biochemical, or radiological signs of cirrhosis were present; the diagnosis of cirrhosis was made solely by histological findings.

All clinical and biochemical parameters were collected at the time of the liver biopsy. The biochemical analysis included hemocrome, ALT, AST, GGT, total bilirubin, blood glucose, triglycerides, total cholesterol, and high-density lipoprotein (HDL) cholesterol. All plasma samples were stored at −80 °C for the investigations.

The study was carried out according to the principles of the Declaration of Helsinki, and it was approved by the ethics committee of the University Hospital “Città della Salute e della Scienza” of Torino (CEI/522, 23 December 2009). All patients gave signed consent for the collection of personal data in the database and for the use of blood samples for research purposes and for participation in the tracer study.

### 4.2. Analytical Determination

Serum eNAMPT was evaluated with a commercially available sandwich enzyme-linked immunosorbent assay for human NAMPT (ELISA kit from AdipoGen Inc; Seoul, Korea) according to the manufacturer’s protocol. Serum eNAPRT was evaluated with a commercially available sandwich enzyme-linked immunosorbent assay for human NAPRT (ELISA kit from Abbexa Ltd.; Cambridge, UK).

### 4.3. Histology

All liver biopsies were examined by a pathologist with expertise in liver diseases and blinded to clinical information. All liver specimens contained at least 11 portal tracts with an average length of 25 mm. The histological features of NAFLD, including steatosis, lobular inflammation, ballooning, and fibrosis, were assessed and staged according to the Clinical Research Network Scoring System (NAFLD Activity Score) [26]. Diagnosis of NASH was made by the joint presence of steatosis, lobular inflammation, and ballooning. Advanced fibrosis was defined by fibrosis stages three to four.

### 4.4. Non-Invasive Assessment of Liver Fibrosis

Liver stiffness was assessed by vibration-controlled transient elastography (Fibroscan, Echosens, Paris, France) at a fasting condition within one week from the liver biopsy. All examinations were performed by an expert operator using the M or XL probe as appropriate. A minimum of 10 measurements were taken for each patient, and technical reliability was assessed by IQR/med ratio < 30%.

The FIB-4 score was calculated according to the following formula [27]: (age(years) × AST(IU/L))/(platelet count(10^9^/L) × rad(ALT(IU/L)).

### 4.5. Statistical Analysis

Data are reported as the median [IQR] for continuous variables and as the frequency (%) for categorical variables. Comparisons between the two groups were made by the Mann–Whitney test. Univariate and multiple stepwise logistic regression analyses were performed to assess the association with advanced fibrosis and to build a model for the prediction of advanced fibrosis, respectively. The accuracy of the eNAPRT, LS, and FIB-4 scores for the identification of advanced fibrosis was assessed using ROC analysis. The sensitivity, specificity, positive predictive value, and negative predictive value were reported on the basis of Youden index-derived cut-offs. The comparison between AUCs was performed using the DeLong test. *p* values < 0.05 were considered statistically significant. All the analyses were performed with the MedCalc Software bvba version 18.9.1 (Mariakerke, Belgium).

## Figures and Tables

**Table 1:** Characteristics of the study cohort.

Parameter (n = 180)	
Male sex, n (%)	114 (63.3)
Age (years), median [IQR]	47.0 [38.0–58.0]
BMI (kg/m^2^), median [IQR]	29.2 [26.3–32.9]
Type 2 diabetes, n (%)	50 (27.8)
AST (IU/L), median [IQR]	31.0 [21.5–43.5]
ALT (IU/L), median [IQR]	48.0 [33.0–76.0]
GGT (IU/L), median [IQR]	47.0 [28.0–97.5]
Total bilirubin (mg/dL), median [IQR]	0.7 [0.5–0.9]
Platelet count (10^9^/L), median [IQR]	230.5 [196.5–274.0]
Glucose (mg/dL), median [IQR]	93.0 [85.5–109.5]
Triglycerides (mg/dL), median [IQR]	124.0 [91.0–175.5]
Total cholesterol (mg/dL), median [IQR]	185.5 [162.0–211.0]
HDL cholesterol (mg/dL), median [IQR]	47.0 [39.0–55.0]
Liver stiffness (kPa), median [IQR]	7.4 [5.8–10.0]
FIB-4 score, median [IQR]	0.9 [0.6–1.3]
eNAMPT (ng/mL), median [IQR]	4.1 [1.8–8.2]
eNAPRT (ng/mL), median [IQR]	2.1 [0.9–5.1]

Abbreviations. ALT: alanine aminotransferase; AST: aspartate aminotransferase; BMI: Body Mass Index; FIB-4: Fibrosis-4; GGT: gamma glutamyl transferase; HDL: high-density lipoprotein; eNAMPT: extracellular nicotinamide phosphoribosyltransferase; eNAPRT: extracellular nicotinate phosphoribosyltransferase.

**Table 2:** Histology findings.

Parameter (n = 180)	
Steatosis, n (%)	
- Grade 1 - Grade 2 - Grade 3	87 (48.3) 64 (35.6) 29 (16.1)
Lobular inflammation, n (%)	
- Grade 0 - Grade 1 - Grade 2	33 (18.3) 130 (72.2) 17 (9.4)
Ballooning, n (%)	
- Grade 0 - Grade 1 - Grade 2	27 (15.0) 96 (53.3) 57 (31.7)
Fibrosis, n (%)	
- Stage 0 - Stage 1 - Stage 2 - Stage 3 - Stafe 4	46 (25.6) 52 (28.9) 27 (15.0) 35 (19.4) 20 (11.1)
Advanced fibrosis (stage 3–4), n (%)	55 (30.6)
NASH, n (%)	140 (77.8)

Abbreviations. NASH: non-alcoholic steatohepatitis.

**Table 3:** Univariate logistic regression analysis for advanced fibrosis.

Parameter	OR	95% Confidence Interval	*p* Value
eNAPRT (ng/mL)	1.08	1.01–1.15	0.016
Liver stiffness (kPa)	1.33	1.20–1.49	<0.0001
FIB-4 score	2.40	1.44–3.97	<0.0001

Abbreviations. FIB-4: fibrosis-4; NAPRT: nicotinate phosphoribosyltransferase.

**Table 4:** Receiving operator curve analysis for advanced fibrosis.

Parameter	AUC	Cut-off (Youden Index)	Se (%)	Sp (%)	PPV (%)	NPV (%)
eNAPRT (ng/mL)	0.60	2.76	56.4	64.0	40.8	76.9
Liver stiffness (kPa)	0.79	9.4	63.5	86.2	66.0	84.8
FIB-4 score	0.73	0.86	80.0	56.8	44.9	86.6

Abbreviations. AUC: area under the curve; FIB-4: fibrosis-4; eNAPRT: extracellular nicotinate phosphoribosyltransferase; NPV: negative predictive value; PPV: positive predictive value; Se: sensitivity; Sp: specificity.

**Table 5:** Multiple stepwise logistic regression analysis for advanced fibrosis.

		Univariate			Multivariate	
Parameter	OR	95% Confidence Interval	*p* Value	OR	95% Confidence Interval	*p* Value
eNAPRT > 2.76 ng/mL	2.24	1.16–4.33	0.015	2.73	1.20–6.16	0.015
Age (years)	1.05	1.02–1.09	0.0001	1.03	1.01–1.07	0.049
Type 2 diabetes	4.28	2.13–8.58	<0.0001	3.49	1.48–8.21	0.004
Male sex	2.65	1.38–5.10	0.003	2.84	1.63–9.05	0.002
ALT (IU/L)	1.01	1.00–1.05	0.050	1.01	1.00–1.02	0.021
AST (IU/L)	1.01	0.99–1.02	0.052	-		
BMI (kg/m^2^)	1.08	1.01–1.16	0.018	-		
GGT (IU/L)	1.00	0.99–1.00	0.891	-		
Total cholesterol (mg/dL)	0.99	0.99–1.00	0.658	-		
HDL cholesterol (mg/dL)	1.01	0.98–1.04	0.257	-		
Triglycerides (mg/dL)	0.99	0.99–1.00	0.361	-		

Abbreviations. ALT: alanine aminotransferase; AST: aspartate aminotransferase; BMI: Body Mass Index; GGT: gamma glutamyl transferase; HDL: high-density lipoprotein; eNAPRT: extracellular nicotinate phosphoribosyltransferase.

## Data Availability

The data presented in this study are available on request from the corresponding author.

## Supplementary Materials

The supporting information can be downloaded at: https://www.mdpi.com/article/10.3390/ijms24021172/s1.
