# Supplementary material for: The Predictive Role of Extracellular NAPRT for the Detection of Advanced Fibrosis in Biopsy-Proven Non-Alcoholic Fatty Liver Disease
Source: Int J Mol Sci. 2023 Jan 7;24(2):1172. doi: 10.3390/ijms24021172 (PMC9861383; doi:10.3390/ijms24021172)
Supplement: Supplementary file 1 [file ijms-24-01172-s001.zip › ijms-2136511-supplementary.pdf]

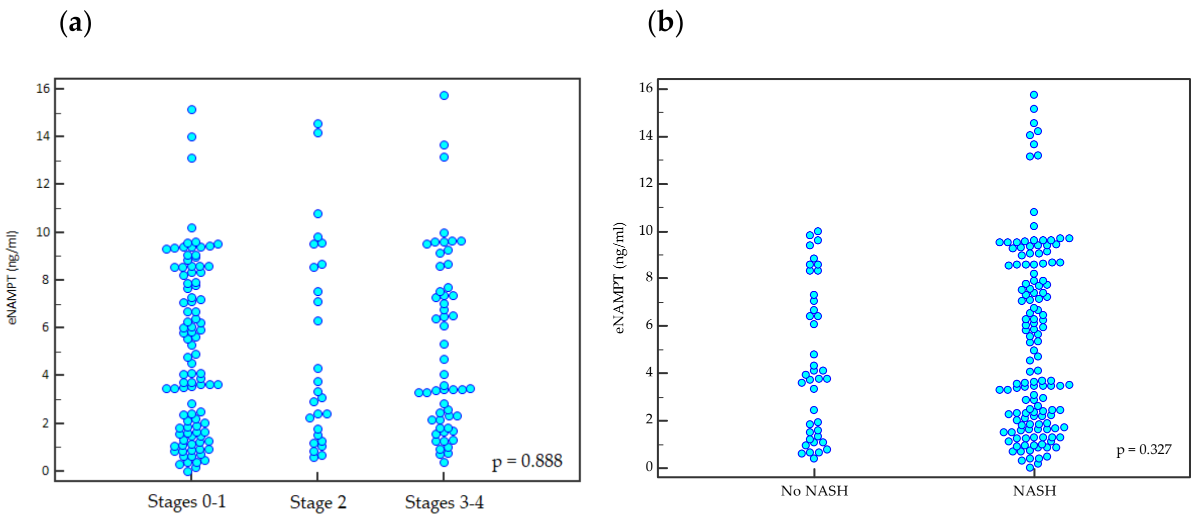

Supplementary Figure S1. Distribution of serum nicotinamide phosphoribosyltransferase (NAMPT) according to fibrosis stages (**a**) and to presence of NASH (**b**).
